# Integrative information theoretic network analysis for genome-wide association study of aspirin exacerbated respiratory disease in Korean population

**DOI:** 10.1186/s12920-017-0266-1

**Published:** 2017-05-24

**Authors:** Sehee Wang, Hyun-hwan Jeong, Dokyoon Kim, Kyubum Wee, Hae-Sim Park, Seung-Hyun Kim, Kyung-Ah Sohn

**Affiliations:** 1 Department of Software and Computer Engineering, Ajou University, Suwon, 16499 South Korea; 2 Jan and Dan Duncan Neurological Research Institute at Texas Children’s Hospital, Houston, Texas 77030 USA; 3 Department of Human and Molecular Genetics, Baylor College of Medicine, Houston, Texas 77030 USA; 4 Department of Biomedical & Translational Informatics, Geisinger Health System, Danville, PA 17822 USA; 5 The Huck Institutes of the Life Sciences, Pennsylvania State University, University Park, PA USA; 6 Department of Allergy and Clinical Immunology, Ajou University School of Medicine, Suwon, South Korea; 7 Translational Research Laboratory for Inflammatory Disease, Clinical Trial Center, Ajou University Medical Center, Suwon, South Korea

**Keywords:** Aspirin exacerbated respiratory disease (AERD), Asthma, Mutual information (MI), Information gain (IG), Genome-wide association study (GWAS), Epistasis, Integrated network, Single nucleotide polymorphisms (SNP)

## Abstract

**Background:**

Aspirin Exacerbated Respiratory Disease (AERD) is a chronic medical condition that encompasses asthma, nasal polyposis, and hypersensitivity to aspirin and other non-steroidal anti-inflammatory drugs. Several previous studies have shown that part of the genetic effects of the disease may be induced by the interaction of multiple genetic variants. However, heavy computational cost as well as the complexity of the underlying biological mechanism has prevented a thorough investigation of epistatic interactions and thus most previous studies have typically considered only a small number of genetic variants at a time.

**Methods:**

In this study, we propose a gene network based analysis framework to identify genetic risk factors from a genome-wide association study dataset. We first derive multiple single nucleotide polymorphisms (SNP)-based epistasis networks that consider marginal and epistatic effects by using different information theoretic measures. Each SNP epistasis network is converted into a gene-gene interaction network, and the resulting gene networks are combined as one for downstream analysis. The integrated network is validated on existing knowledgebase of DisGeNET for known gene-disease associations and GeneMANIA for biological function prediction.

**Results:**

We demonstrated our proposed method on a Korean GWAS dataset, which has genotype information of 440,094 SNPs for 188 cases and 247 controls. The topological properties of the generated networks are examined for scale-freeness, and we further performed various statistical analyses in the Allergy and Asthma Portal (AAP) using the selected genes from our integrated network.

**Conclusions:**

Our result reveals that there are several gene modules in the network that are of biological significance and have evidence for controlling susceptibility and being related to the treatment of AERD.

## Background

Aspirin Exacerbated Respiratory Disease (AERD) is a chronic medical condition that is also called Aspirin-induced Asthma (AIA). Asthma, nasal polyposis, and hypersensitivity reactions to aspirin and non-steroidal anti-inflammatory drugs (NSAIDs) were referred to as AERD [1]. Many previous studies have been conducted to identify genetic variants that affect AERD and related disease [2–8], which revealed that some genetic effects for the disease may be induced by the interaction of multiple genetic variants. However, heavy computational cost as well as the complexity of the underlying biological mechanism has prevented thorough investigation of epistatic interactions [9]. Thus, most previous studies have typically considered only a small number of genetic variants at a time.

Many previous studies proposed different methods to detect high-order genetic interactions using machine-learning approaches or heuristic algorithms [10–17]. However, most of the methods have a limitation that they can detect multi-order interactions only with a small number of SNPs or genes [18]. Furthermore, considering high-order interaction is not typically feasible for the GWAS dataset [19]. In the network analysis, many studies use information theoretic measures such as mutual information or information gain to obtain the strength of association between a pair of SNPs and a trait and then to construct an epistasis network. An extension of relevance network by Butte and Kohane, which uses mutual information with permutation test [18], was applied to a genome-wide data of Korean population and identified potential gene-gene interaction factors that affect the susceptibility to gastritis [19]. McKinney et al. proposed GAIN (Genetic Association Interaction Network) methods, which constructs an interaction network using information gain with SNP prioritization with Evaporative Cooling [20]. Davis et al. extended the McKinney et al.’s work using a network eigenvector centrality algorithm (SNPRank), which is analogous to Google PageRank algorithm to detect interactions that have the weak effect [21], and GAIN with a generalized linear model (reGAIN) was also proposed [22]. Hu et al. proposed SEN (Statistical Epistasis Networks), which use the difference of the number of largest connected components between random networks for network selection, demonstrated the method on bladder cancer [23]. Also, they extended the work that characterizes genes using dyadicity and heterophilicity analyses [24]. Although different methods have shown to be effective for different purposes, few studies enabled systematic and efficient analysis of interacting gene modules in a gene-based network framework given a GWAS dataset.

In this study, we propose a gene network based analysis framework to identify multiple genetic risk factors associated with the disease from a genome-wide association study dataset. We construct SNP-based epistasis networks using different information theoretic measures of mutual information and information gain. Mutual information and information gain mainly consider marginal and epistatic effects, respectively, and can give complementary information. We convert the SNP epistasis networks into gene-gene interaction networks by using the SNPs mapped to each gene and the association strengths of the corresponding pairs of SNPs. Our method improves the previous study [19] for the conversion and gets rid of the dependency of the resulting edge weight on the gene size. We integrate the resulting two gene networks as one to have a better view on the interaction mechanism. We validate our method using existing knowledge databases of DisGeNET [25] for known gene-disease associations and GeneMANIA [26] for biological interaction between genes.

We demonstrate our proposed method on a Korean GWAS dataset, which has genotype information of 440,094 SNPs for 435 unrelated Korean patients. The genotypic data and clinical information of the patient were previously collected with written informed consent and with the approval of the Ethics Review Board of the Ajou University Hospital (AJIRB-GEN-GEN-11-304) in the genome-wide association study of AERD [27]. The SNP data were anonymized and then used for an epistasis analysis for this study. The topological properties of the generated networks are examined for identifying statistically significant edges. For further validation, pathway and gene ontology enrichment tests are performed in the Allergy and Asthma Portal (AAP). AAP is built upon InnateDB [28] that is a previously developed integrated analysis platform for innate immune responses.

## Methods

### Data pre-processing

Our raw dataset consists of 440,094 SNPs from 188 Aspirin Exacerbated Respiratory Disease (AERD) samples and 247 Aspirin Tolerant Asthma (ATA) samples. We use AERD samples as cases and ATA as controls. We filter out SNPs with missing values in more than one-third of the samples. A linkage based imputation method [29] is then applied to remaining missing values. We also remove SNPs with minor allele frequency < 0.05. The resulting dataset consists of 320,815 SNPs.

### Overview

**Fig. 1:**
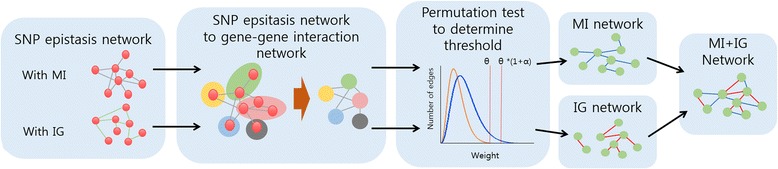
Illustration of the overall process of the proposed gene network based framework

We first give a brief introduction to the overall process of our proposed analysis framework. Figure 1 illustrates each step of the whole analysis process. First, single nucleotide polymorphisms (SNP)-based epistasis networks are constructed by using information-theoretic measures of mutual information (MI) and information gain (IG), respectively, as association measures between a pair of SNPs and the disease. Second, each SNP epistasis network is converted into a gene-gene interaction network. We experiment with different conversion methods and choose the one that is robust to gene size variation. Each converted network is further cut off with an appropriate threshold for edge weights obtained from permutation strategy. The resulting two gene networks are combined as one for downstream analysis. Details of each step are described in the following sections.

### Construction of SNP epistasis networks using information-theoretic measures

We construct SNP epistasis networks in which nodes represent the SNPs, and the edge weight is defined as the association strength between a pair of SNPs and the disease. We consider two information-theoretic measures of mutual information and information gain as for defining the edge weights. Mutual information is a non-parametric measure that represents the amount of information obtained about one random variable through the other [30]. It has been used to detect an association between two random variables [12, 19]. Mutual information of two random variables *X* and *Y* is defined as:

$$ I\left( X; Y\right)= H(X)+ H(Y)- H\left( X, Y\right) $$

where *H*(*X*) and *H*(*Y)* denote the entropy of *X* and *Y*, respectively, and *H*(*X,Y*) is the joint entropy of *X* and *Y*. Mutual information to measure the strength of association between a pair of variables *X*_1_, *X*_2_ and *Y* can be written as follows:

$$ I\left({X}_1,{X}_2; Y\right)= H\left({X}_1,{X}_2\right)+ H(Y)- H\left({X}_1,{X}_2, Y\right) $$

In this study, *X*_1_ and *X*_2_ are discrete random variables for representing two SNPs and *Y* denotes the discrete random variable for the disease label.

While mutual information is largely affected by the marginal effect of either SNP, the information gain [31] mainly reflects the synergistic effect by subtracting each marginal effect of *X*_1_ and *X*_2_ from the mutual information [32] as follows.

$$ I G\left({X}_1;{X}_2; Y\right)= I\left({X}_1,{X}_2; Y\right)- I\left({X}_1; Y\right)- I\left({X}_2; Y\right) $$

Therefore, mutual information and information gain can capture different types of interaction mechanisms. Since the two measures can give complementary information, we construct two different networks, compare the major characteristics, and integrate the two for the final downstream analysis.

### Gene-gene interaction network construction from SNP epistasis network

**Fig. 2:**
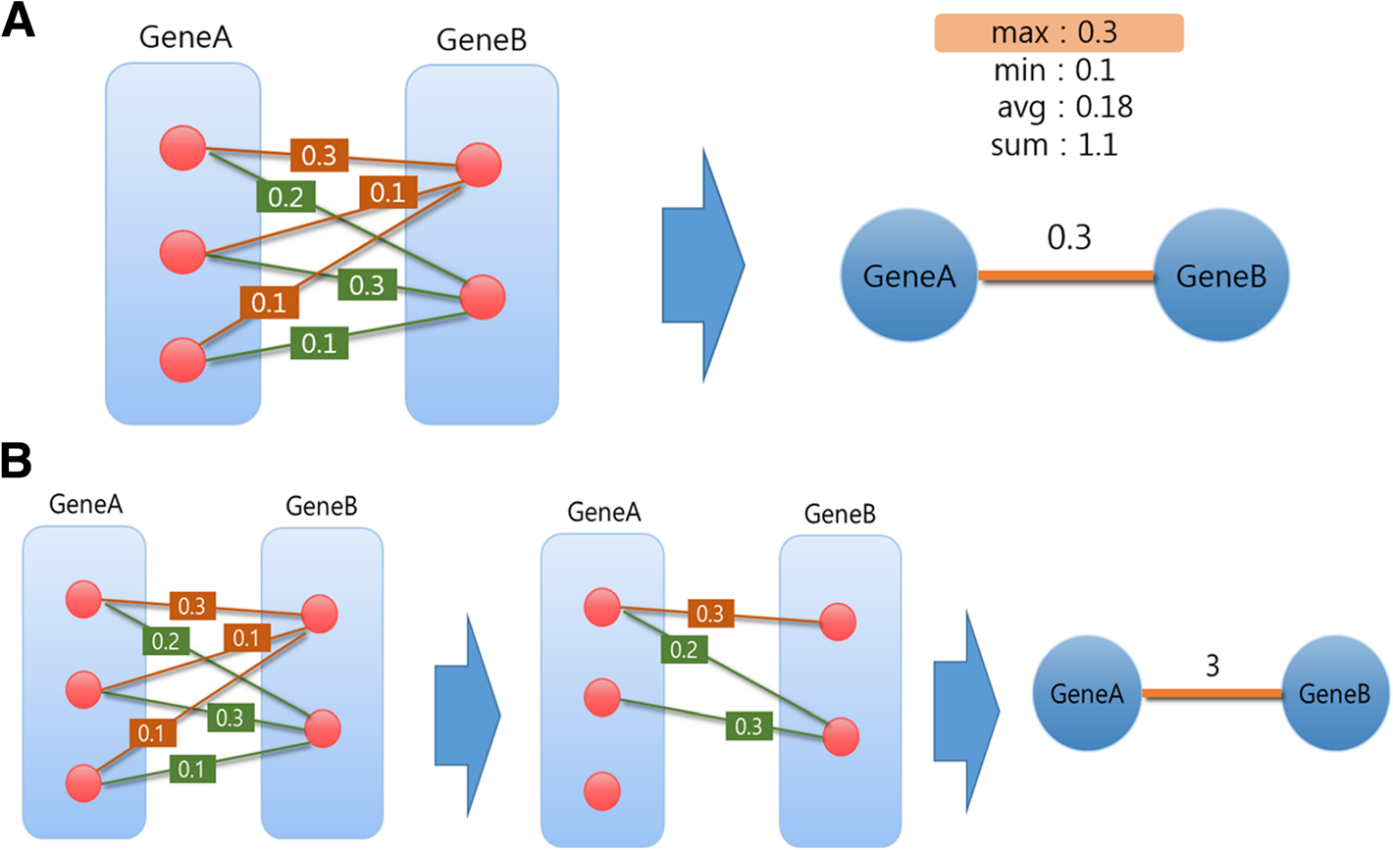
Illustration of the conversion process from a SNP epistasis network to a gene-gene interaction network of our method (**a**) and the one in a previous study [19] (**b**). In this figure, red circles represent the SNP and edge weight is the association strength of two SNPs

To expand the analysis scope from SNPs to genes and enable better interpretation and functional validation in a network framework, we convert the constructed SNP epistasis networks into gene-gene interaction networks. Edge weights of the gene-gene interaction network are computed using the edge weights of SNP epistasis network. As multiple SNPs can be mapped to the same gene, we need an algorithm to determine the weight between two genes given the mapped SNPs and the association strengths between them. Given multiple edge weights between SNPs belonging to two different genes, one may choose different summary statistics as the weight in a gene network such as the sum, average, minimum, or the maximum. Figure 2(a) shows an example of assigning the edge weight of a gene network given SNP epistasis network using different statistics. The summation method suffers from the bias for a longer gene accumulating higher edge weights because more SNPs tend to be mapped to the gene. In contrast, the average method is found to be limited in that the genes having only a couple SNPs tend to have higher degree: if a certain gene has many SNPs in it, it is more likely to contain some SNPs with very low edge weights, and this can substantially affect the average that is sensitive to outliers. The same problem arises in the case of taking the minimum. The maximum method does not suffer from these problems, and the maximum weight can represent the most meaningful interaction between SNPs. So we choose to take the maximum value in the conversion process.

In a previous work [19] that performs similar network analysis, the SNP epistasis network is first cut off by a threshold obtained from a permutation strategy, and then the number of remaining edges in the SNP epistasis network was used to construct a gene-gene network as illustrated in Fig. 2(b). Finally, the top 5% edges with largest weights are chosen for further analysis. In this scheme, the network thresholding is performed twice, one for the SNP network and the other for the converted gene network. Therefore, one needs to define the cut-off each time. Moreover, as it counts the number of SNP pairs mapped to the corresponding genes, it also has the bias with respect to the gene size. That is, long genes that have many SNPs may become hub genes with a high degree even if they are not high risk factors for the disease. In our method, we directly utilize the edge weight in the SNP network instead of counting the number of edges, and also the thresholding is performed only once for the converted gene network as described in Fig. 2.

We also measured the correlation between gene size and node degree to see if our method and previous method were biased by the gene size. The results are described in Results and Discussion section.

### Extraction of a statistically significant interaction network

From the converted gene network, we extract statistically significant edges using a permutation strategy used in a previous study [19] that is similar with the one in [18]. For every network edge, we permute the disease label in the dataset 30 times and calculate the average of 30 permuted edge weights. The maximum of the resulting edge weights is chosen as a network threshold θ. The edges with weights above θ can be regarded as significant interactions.

However, the resulting network can have a huge number of edges such as over one hundred million, which makes the analysis process too slow or even infeasible. To allow systematic adjustment of the number of edges in the network, we incorporate another parameter α and test varying thresholds in the form of θ*(1 + α) as used in MINA [33]. We vary α by 0.1 and choose the most appropriate network by using its topological properties. We also refer to the gene set known to be involved in the disease.

Specifically, we examine the scale-freeness of the constructed networks using an R-square value. Moreover, 1153 genes that are the intersection of 1300 genes related to asthma according to DisGeNET database [25] and the genes in our data are considered as ground truth. With the list of asthma-related genes from the database, we computed a *p-*value that is based on the cumulative distribution function (CDF) of the hypergeometric distribution using our node genes and the ground truth genes in the list. We also calculate Area Under the Curve (AUC) of the network nodes with consideration of the asthma related genes as a ground-truth. For each of mutual information and information gain networks, we select significant edges as described above and examine the network topologies with the major hub genes. Since these two networks can give complementary information on the interaction, we further integrate the two and perform the downstream analysis.

### Validation through prior knowledge databases

**Fig. 3:**
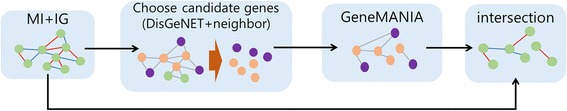
Validation process using DisGeNET and GeneMANIA

We use two external databases to validate our framework. One is DisGeNET [25], a comprehensive discovery platform that is one of the largest repositories currently available of its kind. Another is GeneMANIA [26], a flexible interface used for generating hypotheses about gene functions that provides interactive functional association network. Figure 3 is a graphical illustration of our validation process.

First, we extract genes that are related to asthma from DisGeNET database. Neighbors of them in our integrated network are selected as candidate genes. Then we use this candidate gene list as an input to GeneMANIA to obtain gene-gene interactions that are already identified in previous studies. We compare the resulting interaction network with our MI + IG integrated network to check the overlap as shown in the last step of Fig. 3.

## Results and Discussion

### Network topology

We first examine the network topology of each gene-gene interaction network using mutual information and information gain with a varying threshold of θ*(1 + α) where α is increased by 0.1 and θ is determined through permutation strategy. Tables 1 and 2 summarize the results in the case of mutual information and information gain network, respectively.

**Table 1:** Network topologies for gene-gene interaction network measured by mutual information

Threshold α	Node	Edge	# of component	R^2^ value	AUC	*p-*value
4.7	1762	5022	1	0.594	0.509	3.E-04
4.8	1143	3386	1	0.617	0.542	1.E-02
4.9	690	2328	1	0.671	0.596	4.E-02
5.0	430	1669	2	0.708	0.587	1.E-02
5.1	299	1304	2	0.619	0.565	5.E-03
5.2	213	1047	1	0.685	0.621	2.E-02
5.3	171	858	1	0.654	0.645	2.E-02

**Table 2:** Network topologies for gene-gene interaction network measured by information gain

Threshold α	Node	Edge	# of component	R^2^ value	AUC	*p-*value
3.5	1204	1149	293	0.885	0.508	4.E-02
3.6	827	763	215	0.877	0.542	1.E-01
3.7	526	465	149	0.876	0.542	9.E-02
3.8	333	285	100	0.769	0.504	8.E-02
3.9	227	188	71	0.713	0.456	7.E-02

**Fig. 4:**
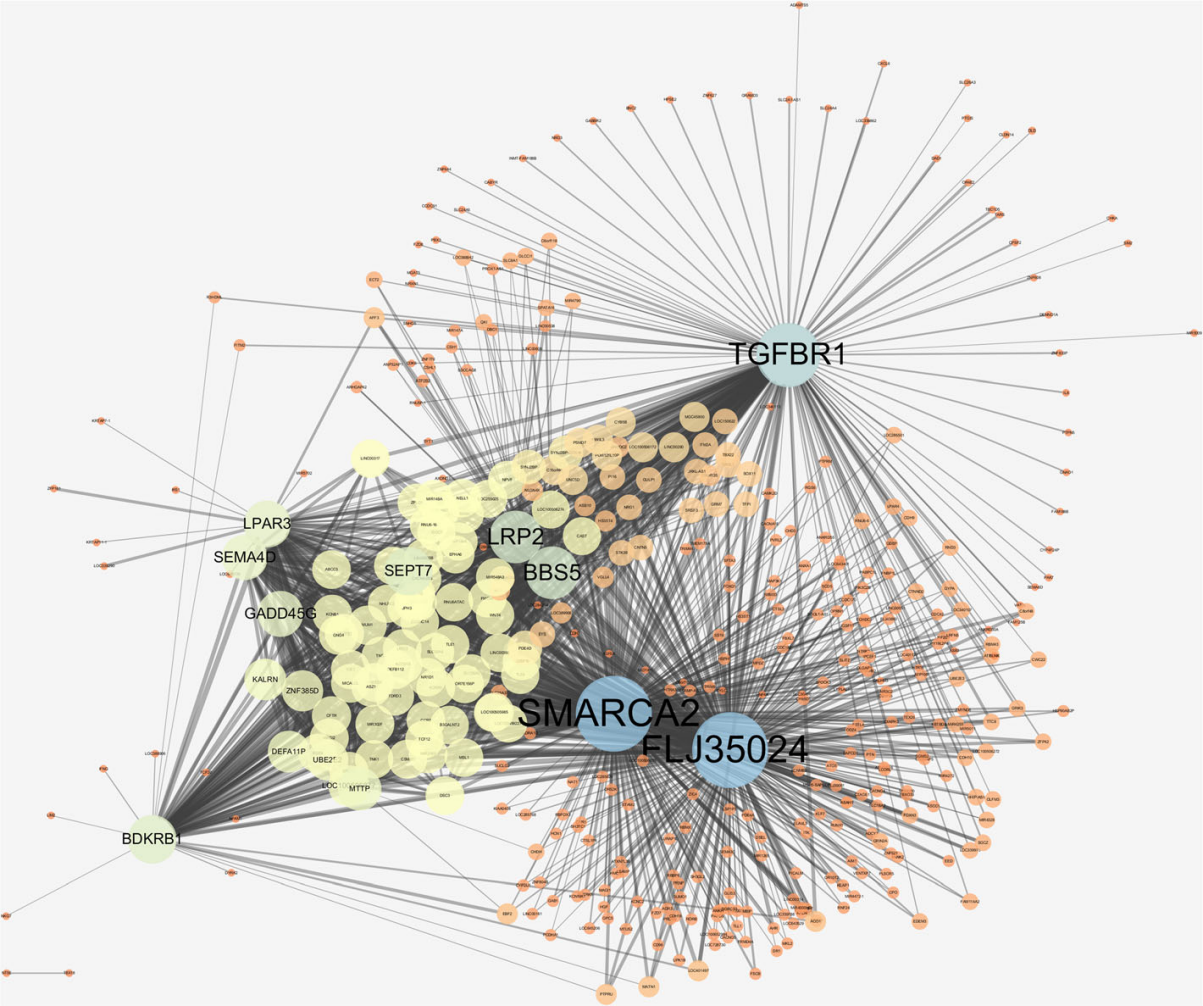
Gene-gene interaction network based on mutual information

In Table 1, we choose the mutual information network with α = 5.0 because it has the local maxima of R^2^ value and the *p-*value from the enrichment test is lower than the conventional significance threshold of 0.05. The chosen network is visualized in Fig. 4. Graphical visualizations of all the networks are produced by using Cytoscape [34]. We find that several hub genes in the network are related to asthma and AERD. For example, in a human study, Transforming Growth Factor Beta Receptor 1 (TGFBR1) has been reported that inhibition of the gene could help treatment of allergy-related conditions, like asthma [35, 36]. The previous study showed that most of Loeys-Dietz syndrome (LDS) patients in the study, who have heterozygous mutations for Transforming Growth Factor Beta Receptor 1 (TGFBR1) or Transforming Growth Factor Beta Receptor 2 (TGFBR2), had been diagnosed with asthma or allergic disease [35, 36]. Moreover, Bradykinin Receptor B1 (BDKRB1), one of the allergic related genes, was reported that the expression of B1 receptor protein for asthma subjects was significantly higher than normal subjects [37]. From the investigation, we presume that the gene-gene interactions of those hub genes and neighbor genes can have an influential role in asthma treatment.

**Fig. 5:**
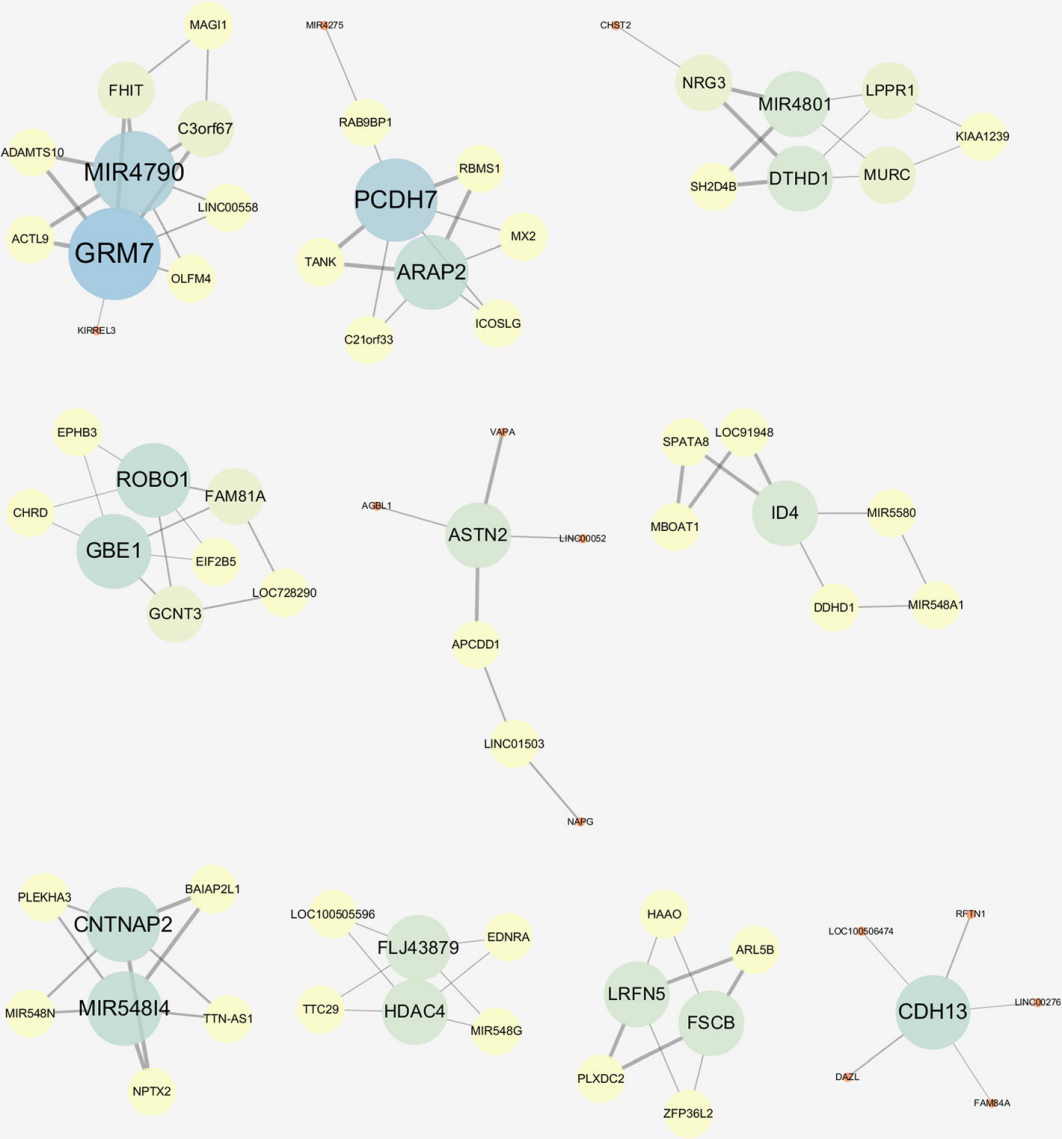
Top-10 largest components of gene-gene interaction network based on information gain

Table 2 summarizes the network properties of the information gain network, from which we choose the network with α = 3.7 that has high R^2^ value, *p-*value from the enrichment test lower than 0.1, and the local maximum of AUC. We observe that the general topology of the information gain network is very different from the one using mutual information. In the case of the mutual information network, there are only one or two connected components across different values of α as shown in Table 1 and illustrated in Fig. 4. In contrast, the information gain network consists of many smaller components as presented in Table 2. We show the top-10 largest components of the chosen network in Fig. 5 as an illustration.

Several hub genes in the network were reported as disease-related in previous studies. Roundabout Guidance Receptor 1 (ROBO1), for example, has been reported as an asthma gene and was differentially expressed during human lung development [38]. Moreover, genetic variants near intragenic region of the gene were reported to have suggestive associations with asthma [39]. Regarding Cadherin 13 (CDH13), lack of its protein (T-cad) has been known to cause reduction of allergic airways disease in the mice study [40]. Existence of strong association between Cadherin 13(CDH13) gene promoter methylation and lung carcinoma risk is also reported [41]. Additionally, we could find that a SNP of Protocadherin 7 (PCDH7) located in flanking 3′ UTR region of the gene, was reported as having significant association in an association study between SNPs and asthma-related quantitative traits [42].

**Fig. 6:**
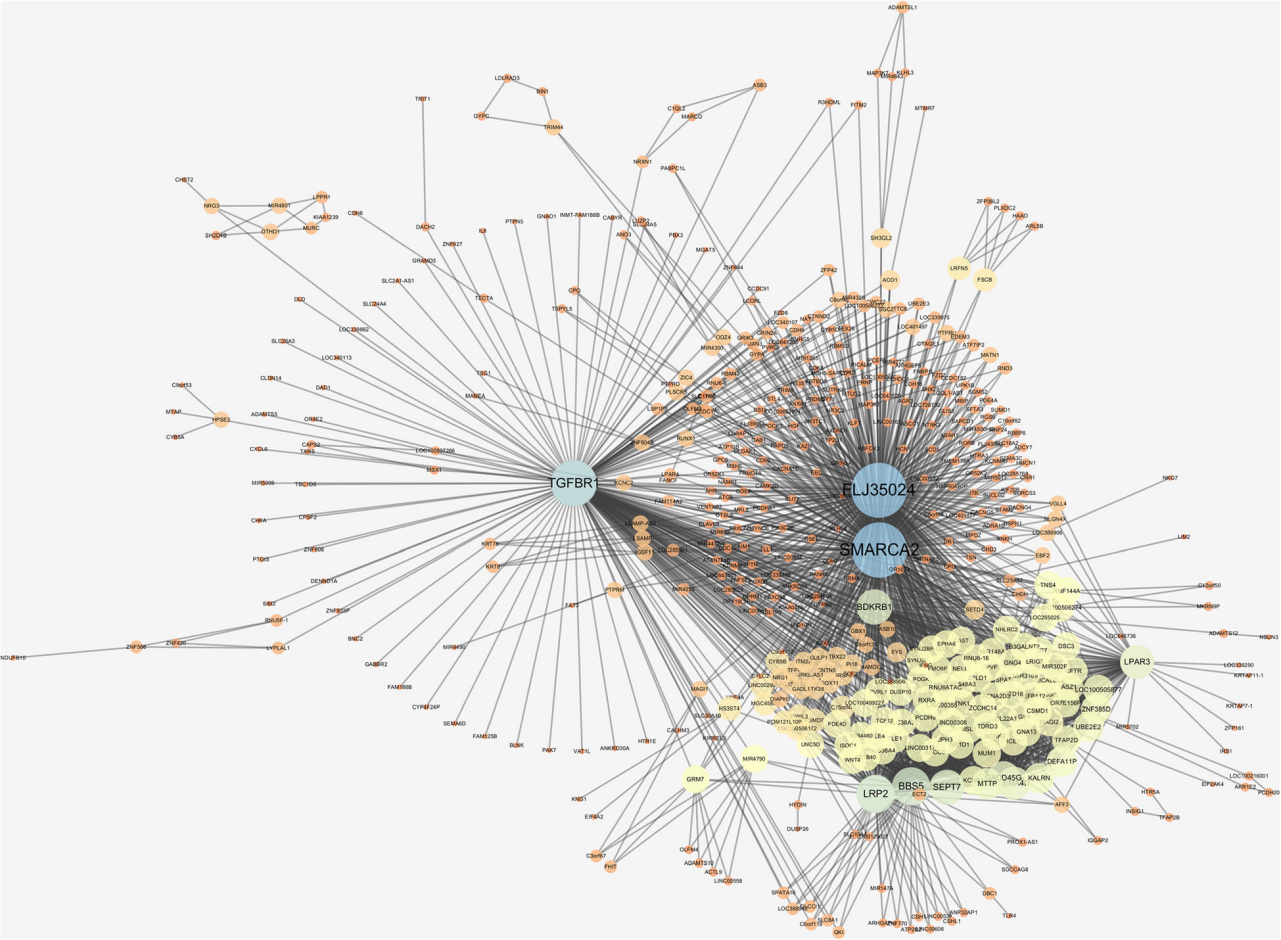
The largest component of MI and IG integrated gene-gene interaction network

The intersection of the two networks from mutual information and information gain produced only one network edge. Since these two networks are highly different and convey different information for the disease, we take the union network as a final network for the downstream analysis. Table 3 shows the network topology of the resulting network. Figure 6 shows the largest component of the integrated network.

**Table 3:** Network topologies for M.I. and I.G integrated gene-gene interaction network

Node	Edge	# of component	R^2^ value	*p-*value
911	2133	120	0.725	7.E-03

### An empirical evaluation of the gene-gene interaction networks for gene length bias

**Fig. 7:**
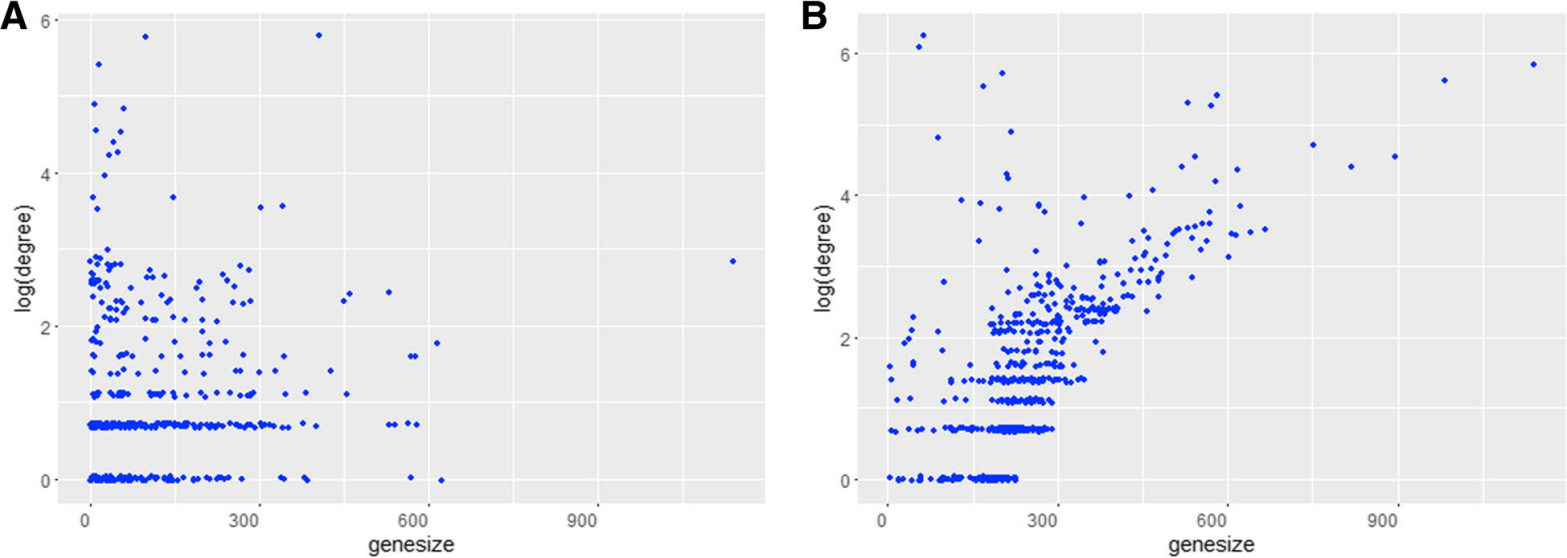
Scatter plots between node degree and gene size of our method (**a**) and previous method [19] (**b**)

We also measured the correlation between gene size and node degree. Figure 7(a) and (b) shows correlation between node degree and gene size of our method and previous method respectively. As we can see previous method is biased by gene size but proposed method is not. The correlation coefficient of previous method is about 0.66, while the correlation coefficient of the proposed method is about 0.04.

### Validation

**Fig. 8:**
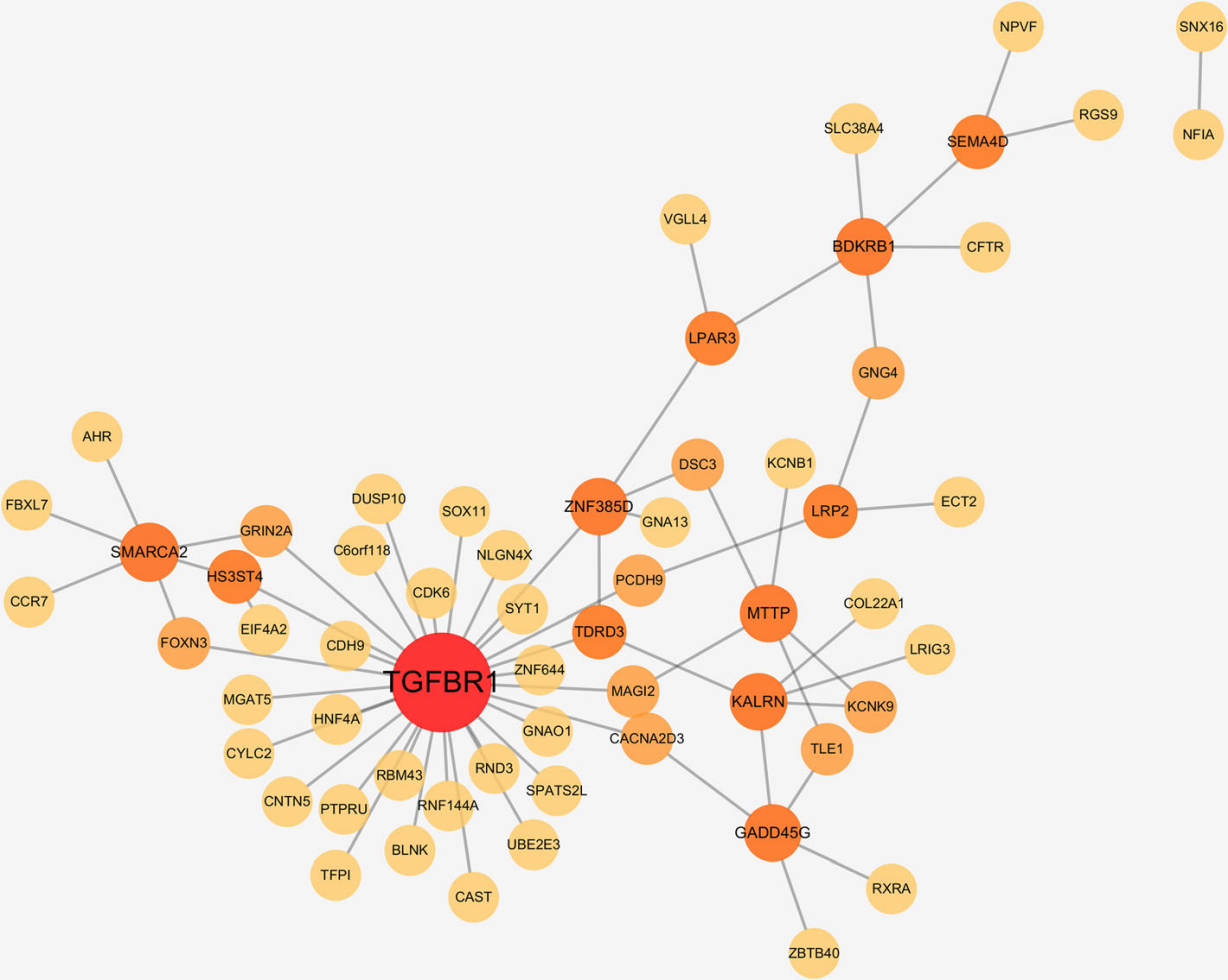
Gene-Gene interaction network constructed using the GeneMANIA Cytoscape plugin. Input genes are intersection of the genes in the network and DisGeNET genes

We first choose 71 genes in our network from DisGeNET data and their neighbor nodes in our network. The resulting 279 genes are used as query genes on GeneMANIA that produces known gene-gene interactions among query genes. The output network from GeneMANIA has 304 nodes and 7725 edges. The intersection of this network with our final network has 279 genes and 67 interactions as shown in Fig. 8, which visualizes those genes that have at least one interaction. In our network, the number of edges between the 279 genes is 820, and the one from GeneMANIA has 6412 edges between 279 genes, so 8.17% edges of our network overlap with the known edges.

### Functional enrichment analysis

We performed pathway and gene ontology enrichment analyses in the Allergy and Asthma Portal using the genes in our final integrated network. The result of pathway enrichment test is shown in Table 4.

**Table 4:** Top 10 pathways having the largest gene count from enrichment analysis (*p-*value < 0.05)

Pathway Name	Pathway Id	Source Name	Gene count	Genes in InnateDB for this entity	*p-*value	*p-*value (corrected)
Signaling by GPCR	17449	REACTOME	30	1035	9.60E-04	4.54E-02
GPCR ligand binding	19266	REACTOME	20	433	1.95E-05	4.61E-03
G alpha (q) signalling events	13217	REACTOME	13	186	8.92E-06	4.22E-03
Gastrin-CREB signalling pathway via PKC and MAPK	13219	REACTOME	13	212	3.60E-05	5.67E-03
G alpha (i) signalling events	13220	REACTOME	13	231	8.71E-05	8.24E-03
Neuroactive ligand-receptor interaction	416	KEGG	13	275	4.84E-04	3.27E-02
GPCR signaling	16218	INOH	13	293	8.76E-04	4.60E-02
Class A/1 (Rhodopsin-like receptors)	13250	REACTOME	13	307	0.001341054	4.88E-02
Transport of inorganic cations/anions and amino acids/oligopeptides	13174	REACTOME	7	94	7.75E-04	4.58E-02
LPA receptor mediated events	15008	PID NCI	6	46	8.69E-05	1.03E-02

In the enrichment analysis of pathways, we can find many modules in the integrated network enrich to G-protein-coupled receptors (GPCR) associated pathways (GPCR ligand binding, Signaling by GPCR, and GPCR signaling), and these pathways can be direct or indirect targets of treatment of AERD or asthma [43]. Several GPCR antagonists have been showed to improve asthma control and reduce exacerbation in clinical trials, e.g. antagonist of P2Y12 (G-protein-coupled purinergic receptor) in a phase II study for AERD treatment [43], antagonist of CRTh2 (G-protein-coupled chemokine receptor homologous molecule expressed on Th2 lymphocytes) in a phase II study for treatment of uncontrolled allergic asthma [44], and antagonist of cysteinyl leukotriene receptor 1 (cysLTR1) for AERD treatment [45]. Furthermore, several genes related to GPCR pathway, such as PDE4A, PDE4D, and ANXA1, were found to be associated with AERD in our data. Drugs that target PDE4 subtypes could play a role in regulating allergic inflammation by attenuating pulmonary eosinophil recruitment, inhibiting lymphocyte proliferation and TFN-α release [46]. Recently, several PDE4 inhibitors have been developed for the treatment of respiratory diseases [47]. Additionally, anti-inflammatory and anti-allergic effects of Annexin A1 have been suggested. Administration of annexin protein prior to an ovalbumin challenge significantly reduced airway hyperresponsiveness, attenuated the production of inflammatory cytokines, such as IL-4, IL-5, and IL-13, as well as ovalbumin-specific IgE in a mouse model of asthma [48]. Meanwhile, neuroactive ligand-receptor interaction, which is another enriched pathway, was reported as a significantly enriched pathway in a differential gene expression study with AERD and ATA subjects [49]. Therefore, we suppose the major modules in the integrated network can play an important role in the pathways related to the treatment of AERD.

Table 5 shows the result of Gene Ontology (GO) enrichment analysis result. From the GO enrichment analysis, we obtain 28 statistical significant terms (after FDR), and most of the statistically significant terms are BP terms (18 terms). We find that several BP terms are related to AERD according to the previous studies. Schäfer and Maune reported that signal transduction (GO:0007165), which showed the second-largest number of associated genes among all BP terms (36 genes), is implicated in AERD and related disease [50]. Several previous studies of the pathogenesis of asthma reported that inflammatory response (GO:0006954) in the airways of asthma patients involves an orchestrated interplay of systems and processes, which drive chronic inflammatory response [51]. Also, another study of AERD pathogenesis reported that the inflammatory disease of AERD is similar to chronic allergic rhinitis and asthma [52].

**Table 5:** Top 10 Gene Ontology terms having the largest gene count from enrichment analysis (*p-*value < 0.05)

GO Term Name	Term Id	Source Name	Gene count	Genes in InnateDB for this entity	*p-*value	*p-*value (corrected)
plasma membrane	GO:0005886	cellular component	85	3645	1.06E-06	1.14E-03
signal transduction	GO:0007165	biological process	36	1368	2.82E-04	2.88E-02
integral component of plasma membrane	GO:0005887	cellular component	29	1063	6.50E-04	4.97E-02
multicellular organismal development	GO:0007275	biological process	20	537	9.77E-05	1.74E-02
signal transducer activity	GO:0004871	molecular function	18	280	1.24E-07	2.66E-04
receptor binding	GO:0005102	molecular function	16	333	2.59E-05	7.92E-03
synaptic transmission	GO:0007268	biological process	15	397	6.26E-04	4.96E-02
dendrite	GO:0030425	cellular component	14	273	4.11E-05	9.77E-03
inflammatory response	GO:0006954	biological process	14	315	1.87E-04	2.36E-02
positive regulation of neuron differentiation	GO:0045666	biological process	8	80	1.87E-05	6.67E-03

## Conclusion

In this study, we presented a gene network based framework for analyzing a genome-wide association study dataset such as SNPs and identifying important risk factors. Although some genetic effects for the disease are induced by the interaction of multiple genetic variants, most previous studies have typically considered only a small number of genetic variants because of heavy computational cost and the modeling complexity. Our proposed method can identify multiple genetic risk factors associated with diseases from a genome-wide association study dataset in a network-based framework. We generate two SNP epistasis networks using mutual information and information gain that convey complementary information. Two SNP epistasis networks are converted to gene-gene interaction networks and finally integrated as one gene-gene interaction network. In the conversion of a SNP epistasis network to a gene network, we handle the bias for long genes accumulating edge weights over multiple mapped SNPs by taking the maximum weight among candidate weights. This can effectively alleviate the problem of previous approaches that can select long genes regardless of whether they are true risk factors or not. The validation method using existing knowledge databases and functional enrichment analysis shows that our framework could identify essential genes associated with the disease.

One limitation of our framework is that we have to set a threshold to cut the network by checking network topologies manually. We calculate threshold θ for significance level and employ an additional parameter α to control the sparsity, but the selection of appropriate threshold is not fully automatic and rather heuristic. We would explore other methods to address this issue in our future work. We also plan to focus on more biological interpretation of the generated networks to find meaningful interactions between multiple genetic variants. Other future work will include the application of the proposed methods to other diseases.

## Abbreviations

AERD: Aspirin exacerbated respiratory disease
GWAS: Genome-wide association study
IG: Information gain
MI: Mutual information
SNP: Single nucleotide polymorphisms
